# The bile acid-sensitive ion channel (BASIC) mediates bile acid-dependent currents in bile duct epithelial cells

**DOI:** 10.1007/s00424-021-02622-2

**Published:** 2021-09-21

**Authors:** Shari Wiegreffe, Daniel Löhrer, Monika Wirtz, Dominik Wiemuth

**Affiliations:** Institute of Physiology, RWTH Aachen University, Pauwelsstrasse 30, 52074 Aachen, Germany

**Keywords:** Cholangiocyte, BASIC, Bile acid, BLINaC, Cation channel, Deg/ENaC

## Abstract

The bile acid-sensitive ion channel (BASIC) is a member of the Deg/ENaC family of ion channels that is activated by bile acids. Despite the identification of cholangiocytes in the liver and unipolar brush cells in the cerebellum as sites of expression, the physiological function of BASIC in these cell types is not yet understood. Here we used a cholangiocyte cell line, normal rat cholangiocytes (NRCs), which expresses BASIC to study the role of the channel in epithelial transport using Ussing chamber experiments. Apical application of bile acids induced robust and transient increases in transepithelial currents that were carried by Na^+^ and partly blocked by the BASIC inhibitor diminazene. Genetic ablation of the BASIC gene in NRC using a CRISPR-cas9 approach resulted in a decrease of the bile acid-mediated response that matched the diminazene-sensitive current in NRC WT cells, suggesting that cholangiocytes respond to bile acids with a BASIC-mediated Na^+^ influx. Taken together, we have identified BASIC as a component of the cholangiocyte transport machinery, which might mediate a bile acid-dependent modification of the bile and thus control bile flux and composition.

## Introduction

The bile acid-sensitive ion channel (BASIC) is probably the least studied mammalian member of the degenerin/epithelial Na^+^ channel (Deg/ENaC) family of ion channels [40]. Deg/ENaCs share several structural, electrophysiological, and pharmacological features [16]. Each channel consists of three subunits, which are composed of two transmembrane domains and linked by a large extracellular domain, N-terminal and C-terminal domains protruding into the cytosol [4, 15]. Deg/ENaCs are voltage-independent and mainly conduct monovalent cations with different selectivities [16]. A common inhibitor of Deg/ENaCs is the diuretic amiloride [16], ASICs and BASIC are also inhibited by diminazene, a diarylamidine that is used as an antiprotozoal drug in veterinary medicine [7, 37].

While the function of two subfamilies within the Deg/ENaC family, the epithelial Na^+^ channel (ENaC) subfamily and the acid sensing ion channels (ASICs) subfamily, is well described [16], very little is known about the physiological role of BASIC. BASIC was originally named brain-liver-intestine Na^+^ channel (BLINaC) as it is predominantly expressed in the brain, liver, and intestinal tract [29]. More detailed analysis revealed expression in unipolar brush border cells of the cerebellum [5] and cholangiocytes, the epithelial cells lining bile ducts in the liver [38]. In UBCs, BASIC seems to be involved in intrinsic excitability [17], and the physiological function of BASIC in cholangiocytes, however, is not understood. Various bile acids activate BASIC robustly and reversibly in heterologous cell systems with various potencies but concentrations in the millimolar range are required for strong activation of BASIC [38, 22, 39]. The most potent effects were observed for hyodeoxycholic acid (HDCA) and ursodeoxycholic acid (UDCA) [39]. It has been proposed that BASIC is sensitive to alterations of its membrane environment and the mechanism of bile acid-dependent BASIC activation is based on a bile acid-induced modification of membrane properties, which in turn leads to an activation of the channel [30]. Concentrations of bile acids that activate BASIC in heterologous systems are reached in bile ducts; hence, a putative bile acid-dependent role for BASIC in the context of an electrolyte modification of the bile fluid can be assumed [38].

Cholangiocytes form the epithelial barrier of bile ducts in the liver. Like other epithelial cells, cholangiocytes are polarized and apical and basolateral plasma membrane regions execute multiple transport functions, which are relevant to bile formation and modification [3, 32]. Cholangiocytes are critical for generation and modification of the bile fluid and thus crucial for one of the liver’s main functions, aiding digestion of fat by providing bile acids to the intestinal tract. Bile is a secretory fluid generated by the hepatobiliary system and contains a variety of components, e.g. bile acids, electrolytes, lipids, proteins, and endobiotic and xenobiotic compounds [3, 32]. Primary bile is generated by hepatocytes and secreted into the canalicular system of the liver. Subsequently, cholangiocytes regulate flow and composition of the bile [3, 32]. One of the main mechanisms of bile flow regulation is the secretion of HCO_3_^−^ to the ductal lumen [13]. This is achieved by an apically located interplay of Cl^−^ secretion mediated by the cystic fibrosis conductance regulator (CFTR) and secretion of HCO_3_^−^ in exchange with Cl^−^ via the anion exchanger 2 (AE2) [2, 8, 10, 26, 33]. This process in turn drives the osmotic secretion of water via aquaporin 1 [25]. It is positively controlled by the hormone secretin [21] and inhibited by various other hormones, like somatostatin, gastrin, and endothelin [6, 11, 12]. Secretion of HCO_3_^−^ leads to the formation of a so-called biliary bicarbonate umbrella, an alkaline barrier that renders bile acids deprotonated and thus polar and membrane impermeable [14, 34]. Furthermore, bile contains several factors that also affect biliary secretion. Extracellular nucleotides activate P2Y receptors at the apical membrane [27], increasing cytosolic Ca^2+^ concentrations and thus enhance Cl^−^ secretion through apical Ca^2+^-activated Cl^−^ channels, such as transmembrane member 16A (TMEM16A; ANO1), a process that promotes HCO_3_^−^ secretion [24]. Absorptive processes also occur in cholangiocytes; e.g., conjugated bile acids can be reabsorbed from the bile via an apical Na^+^-dependent bile salt transporter (ASBT) [18], and glucose can be absorbed by vectorial transport through the apical Na^+^-glucose cotransporter 1 (SGLT1) and the basolateral glucose transporter 1 (GLUT1), which provides an osmotic gradient that favors the reabsorption of water from bile [19]. ENaC is also found in cholangiocytes and reabsorbs Na^+^ upon mechanical stimulation by bile flow [23]. The presence of other transport proteins and ion channels for example P2X receptors [9, 41] suggests further complex regulatory mechanisms in cholangiocyte transport processes.

In this study, we have used a cultured cholangiocyte cell line derived from rat liver in a pharmacological and a gene knockout approach to unravel the physiological role of BASIC in bile duct physiology. We show that bile acids induce desensitizing transepithelial currents, which are partly mediated by BASIC. Thus, our study attributes for the first time a physiological role for BASIC in an epithelial cell type.

## Materials and methods

### Cell culture

Normal rat cholangiocytes (NRC) were a gift from Dr. Nicholas F. LaRusso (Mayo Clinic, College of Medicine, Rochester, Minnesota). Cells were grown on collagen-coated petri dishes (cell coat collagen Typ I, Greiner Bio One, Austria) in cholangiocyte growth medium as described previously [35].

### Immunocytochemistry

Glass coverslips were coated with type I collagen from rat tail (Merck Sigma, Germany) and NRCs were seeded at a density of approximately 5 × 10^5^ cells per 35 mm well and incubated for 13–17 days. NRCs were washed three times with PBS and fixed with PBS containing 4% paraformaldehyde for at least 10 min at room temperature. Cells were washed twice with PBS and permeabilized by incubation with PBS containing 0.3% Triton-X-100 for 20 min at room temperature. Cells were washed three times with PBS and incubated with blocking solution containing 10% normal goat serum for 1 h. Coverslips were transferred to a humidified chamber and incubated with a custom-made polyclonal anti-BASIC antibody from rabbit (Eurogentec, Belgium) (1:100 dilution) in blocking solution overnight at 4 °C. Cells were washed three times with PBS and incubated with AlexaFluor® 488-conjugated anti-rabbit secondary antibody (1:500 dilution) for 1 h at 37 °C. To stain the nuclei, cells were incubated with PBS containing DAPI (1:5000 dilution) for 5 min at RT. After two washing steps, coverslips were dried and mounted in Mowiol (Merck Sigma, Germany). Stained cells were analyzed using a Zeiss LSM 700 confocal microscope (Carl Zeiss, Germany).

### Molecular biology and CRISPR-cas9 knockout strategy

For the ablation of the BASIC gene, a CRISPR-cas9(D10A) paired nickase approach was used [28]. Briefly, two pX335B vectors, each containing two sgRNA coding sequences, were generated (sgRNAs: (1) CCAATATCAATAATTAGCTATGG, (2) GATTGCACTTAGGACTCTTGGGG, (3) CCAACGACAACATCTATAGAAGT, (4) TGTGGAGAAAATTGAGTTCCCGG). For selection of transfected cells, pX335B contains the coding sequence for GFP. Cells were transfected using XtremeGENE9 (Merck Sigma, Germany) and transfected cells were sorted by FACS (Flow Cytometry Facility, a core facility of the Interdisciplinary Center for Clinical Research (IZKF) Aachen within the Faculty of Medicine at RWTH Aachen University). Transfected cells were expanded, genomic DNA was isolated, and successful deletion of exon 2 was detected by PCR (genotyping primer: for TACATCCCCCTTTATTGCC′, rev CCTGTTCAAGTTACAGAATGTCA) and sequencing (Eurofins, Germany). For transcription analysis, mRNA from NRC^BASIC+/+^ and NRC^BASIC−/−^ cells was isolated using the RNeasy Mini Kit and cDNA was synthesized using the QuantiTect Reverse Transcription Kit (Qiagen, Germany) according to the manufacturer’s instructions. Qualitative PCR was performed to show the presence or absence of BASIC and tubulin (control) mRNA (primer: BASIC for GCGAAGAAACAGAATACCCTGC, rev CTGTCTGAGGTGGAGAAGTCC; tubulin for CCAGGGCTTCTTGGTTTTCC, rev CGCTCAATGTCGAGGTTTCT).

### Ussing chamber experiments

NRCs were seeded onto 12-mm Snapwell™ cell culture inserts (Corning Costar, Sigma-Aldrich, St. Louis, Mo, USA) at a density of approximately 5 × 10^5^ cells per Snapwell™ and incubated for 13–17 days. Transepithelial electrical resistance (TEER) of NRC was measured daily using an Ohm voltmeter (Scientific Instruments, Aachen, Germany). Culture medium was exchanged every 48 h and 24 h prior to Ussing chamber measurements. After 13 days and when the TEER was ≥ 400 Ω/cm^2^, epithelia were mounted onto a modified Ussing chamber (Scientific Instruments, Aachen, Germany) stored in a 37 °C incubator. The Ussing chamber was connected to a 6-channel V/A clamp and data were recorded using the software CLAMP (Scientific Instruments, Aachen, Germany) running on a PC. Apical and basolateral surfaces were bathed in 1 × Ringer’s solution (in mM 140 NaCl, 4 KCl, 2.4 KH_2_PO_4_, 1 CaCl_2_, 2 MgCl_2_, 10 glucose, pH 7.4), kept at 37 °C. For low Na^+^ experiments, apical and basolateral surfaces were bathed in 1 × low Na^+^ Ringer’s solution (in mM 2.5 NaCl, 137.5 NMDG, 4 KCl, 2.4 KH_2_PO_4_, 1 CaCl_2_, 2 MgCl_2_, 10 glucose, pH 7.4, adjusted with HCl). Epithelia were measured in open-circuit configuration, transepithelial potential and resistance were measured every 20 s, and transepithelial current was calculated according to Ohm’s law. Peak amplitudes were determined as the difference in current recorded prior to the application of a substance to the apical bathing solution and the maximal current response after the application of a substance.

### Chemicals

Diminazene, amiloride, taurochenodeoxycholic acid (CDCA), taurohyodeoxycholic acid (HDCA), and porcine bile extract (BE) were purchased from Sigma-Aldrich (Germany). Tauroursodeoxycholic (UDCA) acid was purchased from Merck (Germany).

### Data analysis and statistics

Data were analyzed using the software Prism (GraphPad, San Diego, USA) and are presented as means ± SEM. Statistical significance was calculated using Student’s unpaired *t* test.

## Results

### Bile acids induce BASIC-dependent transepithelial currents

**Fig. 1:**
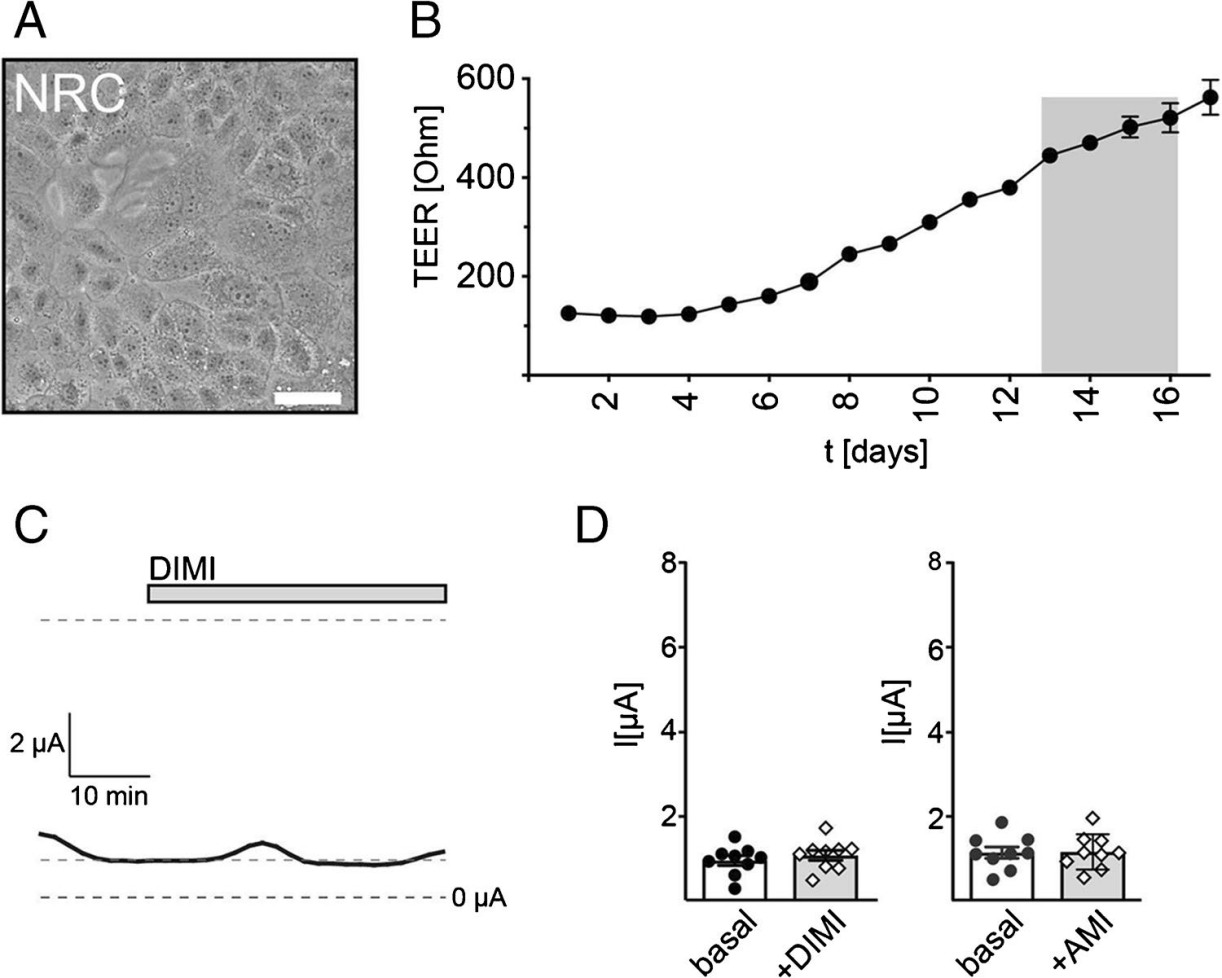
Cultured normal rat cholangiocytes (NRC) display an epithelial-like phenotype and develop a moderate transepithelial electrical resistance. **A** Phase-contrast microscopy shows the epithelial morphology of NRCs grown on cell culture inserts. Scale bar = 20 µm. **B** Average development of the transepithelial electrical resistance (TEER) of NRC epithelial layers over time. Day 0 represents seeding of cells on cell culture inserts. Cells were used for Ussing chamber experiments between day 13 and 16 (labeled in gray), *n* = 63. **C** Representative trace of transepithelial currents recorded from NRCs. Diminazene (DIMI) (10 µM) was applied to the apical lumen at the indicated time. **D** Quantitative comparison of the basal transepithelial currents before and after application of 10 µM DIMI or 10 µM amiloride (AMI), *n* = 9. Error bars = SEM

**Fig. 2:**
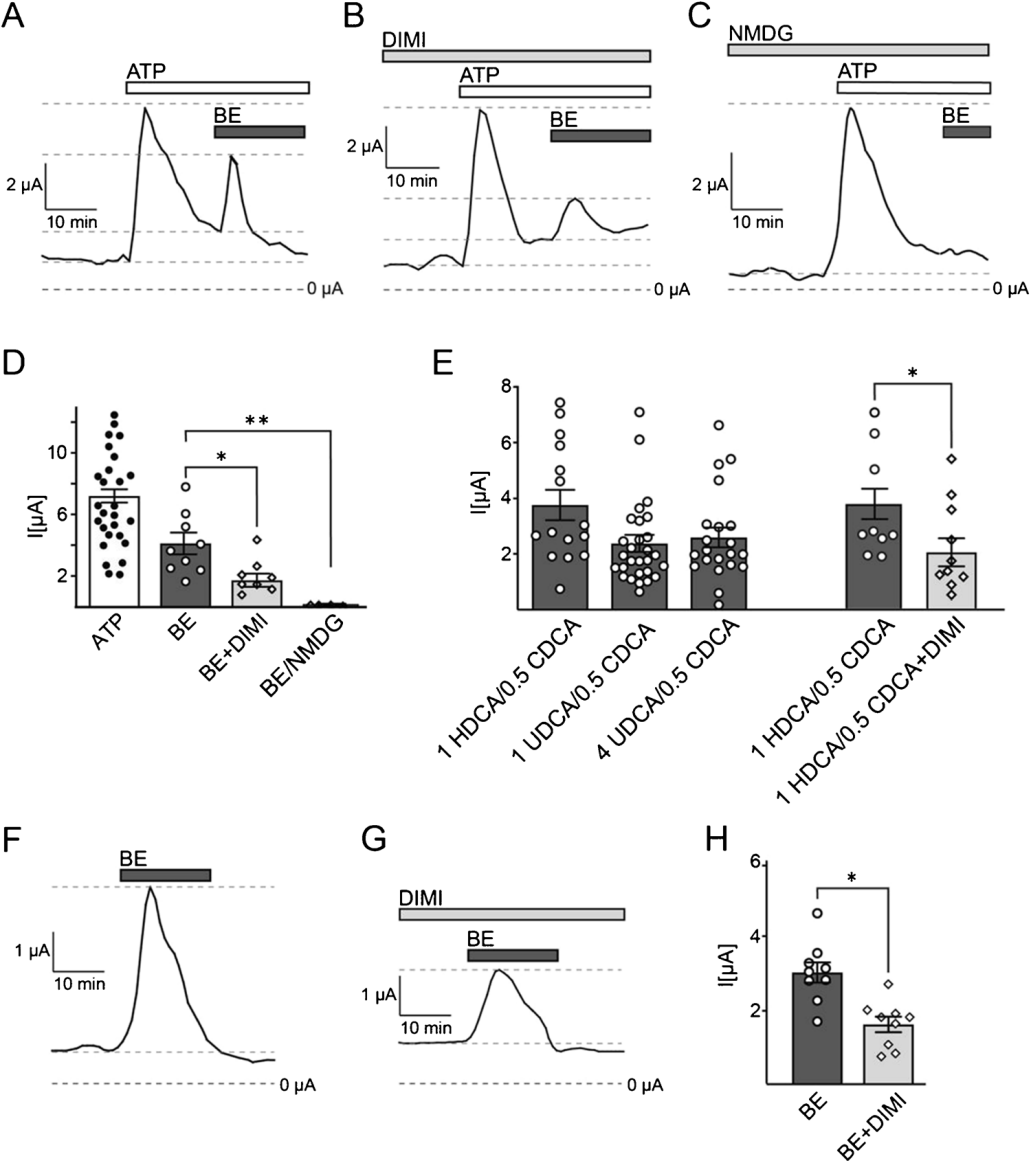
Bile acids induce BASIC-dependent transepithelial currents in NRCs. **A** Representative trace of transepithelial currents recorded from NRCs. ATP (100 µM) and bile extract (BE, 30 mg/ml) were applied to the apical lumen at the indicated times. **B** As in **A** but for NRCs pretreated with 10 µM diminazene. **C** As in **A** but extracellular Na^+^ was replaced by NMDG^+^. **D** Quantitative comparison of the peak amplitudes of the transepithelial currents induced by application of 100 µM ATP or BE in the absence or presence of 10 µM diminazene (DIMI). **E** Quantitative comparison of the peak amplitudes of the transepithelial currents induced by apical application of HDCA/CDCA (1 mM/0.5 mM) or UDCA/CDCA (1 mM/0.5 mM and 4 mM/0.5 mM) or HDCA/CDCA (1 mM/0.5 mM) in the absence or presence of 10 µM DIMI. **F** As in **A** but without application of ATP. **G** As in **B** but without application of ATP. **H** Quantitative comparison of the peak amplitudes of the transepithelial currents induced by application of BE in the absence or presence of 10 µM DIMI. Error bars = SEM, *n* = 8–9, * indicates a significant difference to control at a level of *p* < 0.05 or ** *p* < 0.01

To address whether BASIC is involved in ion transport processes in cholangiocytes, we used normal rat cholangiocytes (NRCs) [35]. NRCs maintained an epithelial-like, cholangiocyte phenotype when grown on cell culture inserts with 0.4-μm pore size coated with rat tail collagen (Fig. 1A) and cells developed a moderate transepithelial electrical resistance (TEER) of approximately 400–600 Ω/cm^2^ after 2 weeks of culturing (Fig. 1B), which is in line with previous results [35]. Once the TEER of the NRC cell culture inserts had reached 400–600 Ω/cm^2^, Ussing chamber experiments were performed to measure transepithelial currents of the NRC epithelial layer in particular bile acid-induced currents. NRC epithelial layers exhibited a basal transepithelial current of 1 ± 0.1 µA, which was insensitive to the BASIC blockers diminazene and amiloride (Fig. 1C/D). Cholangiocytes express purinergic P2 receptors, which stimulate Cl^−^ secretion via TMEM16A [27, 24]. They provide a convenient way to assess the functionality of the NRC epithelium. Application of 100 µM ATP to the apical side of an NRC epithelium typically resulted in a strong increase in transepithelial current of 7.2 ± 0.4 µA (Fig. 2A-D). The peak was reached after 2–3 min and current returned to baseline levels after 12–15 min (Fig. 2A-C). NRC layers not responding to ATP were not further included in experiments. Next, we applied pig bile extract (BE) to the apical lumen at a concentration (30 mg/ml) that is sufficient to strongly activate BASIC in *Xenopus* oocytes [38]. BE induced a strong increase in transepithelial current of 4.1 ± 0.7 µA (Fig. 2A/D). The peak was reached after 1–3 min and current returned to baseline after 5–7 min (Fig. 2A). In control experiments, in which only the vehicle solution was applied, no current increase was observed (data not shown). To test whether the BE-induced stimulation of transepithelial current was mediated by BASIC, the experiment was repeated in the presence of 100 µM diminazene, an inhibitor of BASIC [37] (Fig. 2B/D). While the ATP-mediated response was not affected by diminazene, the BE-induced response was significantly reduced to 1.7 ± 0.4 µA (Fig. 2B/D), indicating that approximately 50% of the BE-induced transepithelial current is indeed mediated by BASIC. BE induced a similar diminazene-sensitive stimulation of transepithelial currents without prior application of ATP, albeit to a weaker extent (3 ± 0.3 µA) (Fig. 2F-H).

Next, we repeated the experiments but applied purified bile acids instead of bile extract. Previous studies had shown that the combination of the two bile acids hyodeoxycholic acid (HDCA) and chenodeoxycholic acid (CDCA) was the most potent activator of BASIC [38, 39]. Therefore, we applied 1 mM HDCA and 0.5 mM CDCA to NRC epithelia. As expected, HDCA/CDCA induced similar responses as the bile extract (3.8 ± 0.6 µA) (Fig. 2E). Ursodeoxycholic acid (UDCA), another potent activator of BASIC, was also co-applied at 1 mM with 0.5 mM CDCA to NRC epithelia and also increased the transepithelial current albeit to a lower extent (2.4 ± 0.3 µA). A further increase in the UDCA concentration to 4 mM did not increase the current further (2.6 ± 0.4 µA) (Fig. 2E), suggesting that a maximal activation of BASIC in NRC was already reached at 1 mM UDCA. Furthermore, we confirmed the inhibition of the BE-induced current increase by diminazene also for the bile acids HDCA/CDCA (Fig. 2E).

BASIC is a cation channel with a slightly higher permeability for Na^+^ over K^+^ [36]. Under the ionic conditions of our experiments, activation of BASIC would mainly lead to Na^+^ influx and thus to transepithelial currents similar to the ATP-induced Cl^−^ efflux. To confirm that the bile acid-induced currents were indeed carried by Na^+^, we replaced the Na^+^ in the apical solution by NMDG^+^, a cation that is not conducted by BASIC [36]. Interestingly the response of the NRC epithelial layer to bile acids was almost completely abolished (Fig. 2C/D), indicating that the bile acid-induced current is solely carried by Na^+^, consistent with a contribution of BASIC.

### Establishing a BASIC-deficient cholangiocyte cell line

**Fig. 3:**
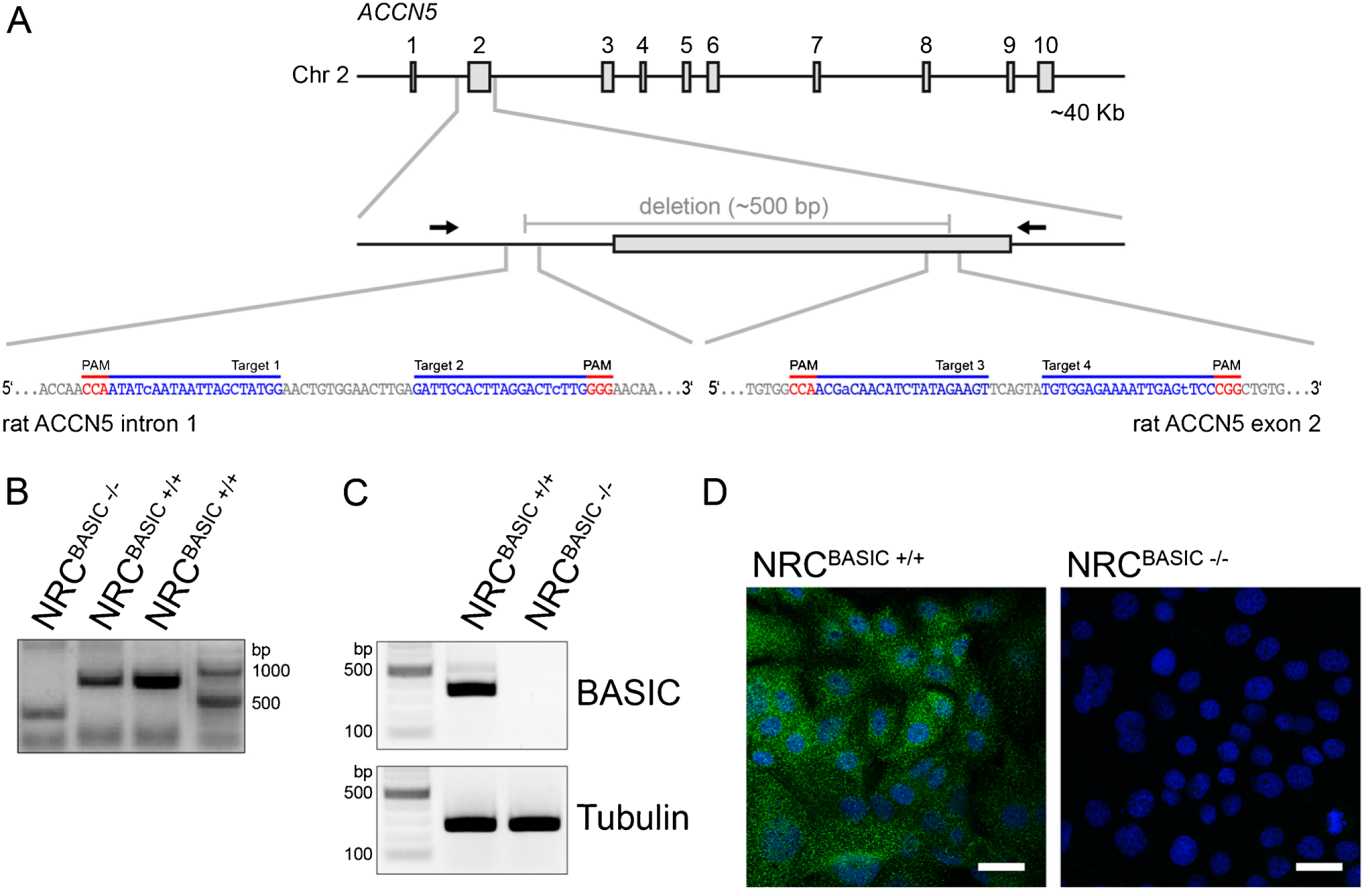
Specific CRISPR Cas9(D10A) paired nickase-mediated knockout of BASIC. **A** Scheme of the CRISPR-Cas9-D10A paired nickase approach for the genomic ablation of BASIC in NRCs, blue: sgRNAs, red: PAM motif, arrows: primer position for genomic PCR. **B** Genomic control PCR showing the deletion of a 500-bp fragment in NRC^BASIC−/−^. **C** RT-PCR showing the absence of BASIC transcript in NRC^BASIC−/−^. **D** Immunofluorescence staining for BASIC in NRC^BASIC+/+^ and NRC^BASIC−/−^. Paraffin-fixed cells were permeabilized and stained with polyclonal rabbit anti-BASIC antibody and AlexaFluor488 secondary anti-rabbit antibody. Blue: nuclei, scale bar: 20 µm

The pharmacological results indicate that BASIC is involved in ion transport processes. We aimed to verify these data using a cholangiocyte cell line, in which the BASIC gene is deleted and no BASIC protein is present anymore. To generate a BASIC knockout NRC cell line, we used the CRISPR-cas9(D10A) gene editing system. We deleted a large portion of exon 2 and an adjacent intron region preceding exon 2 using a paired nickase approach, in which we designed 4 sgRNAs, which direct CRISPR-cas9(D10A) nickase to the regions of interests (Fig. 3A). Paired single strand breaks were induced and subsequently the region between these strand breaks was deleted [28]. As expected, our approach resulted in a 500-bp deletion as verified by genomic PCR (Fig. 3B) and sequencing. Successful knockout of BASIC was verified by RT-PCR. While in NRC WT a specific band of the expected size was present, no BASIC mRNA was detectable in NRC KO cells (Fig. 3C). To further validate the knockout of BASIC, we performed immunostaining of cultured NRCs using a polyclonal anti-BASIC antibody. No expression of BASIC was detected in NRC^BASIC−/−^ cells. In contrast, in NRC^BASIC+/+^ cells, a punctate pattern of BASIC throughout the cytosol was detected (Fig. 3D). In summary, BASIC knockout cells were successfully generated by the CRISPR-cas9(D10A) system targeting exon 2.

### Bile acid-induced currents in cholangiocytes are partly carried by BASIC

**Fig. 4:**
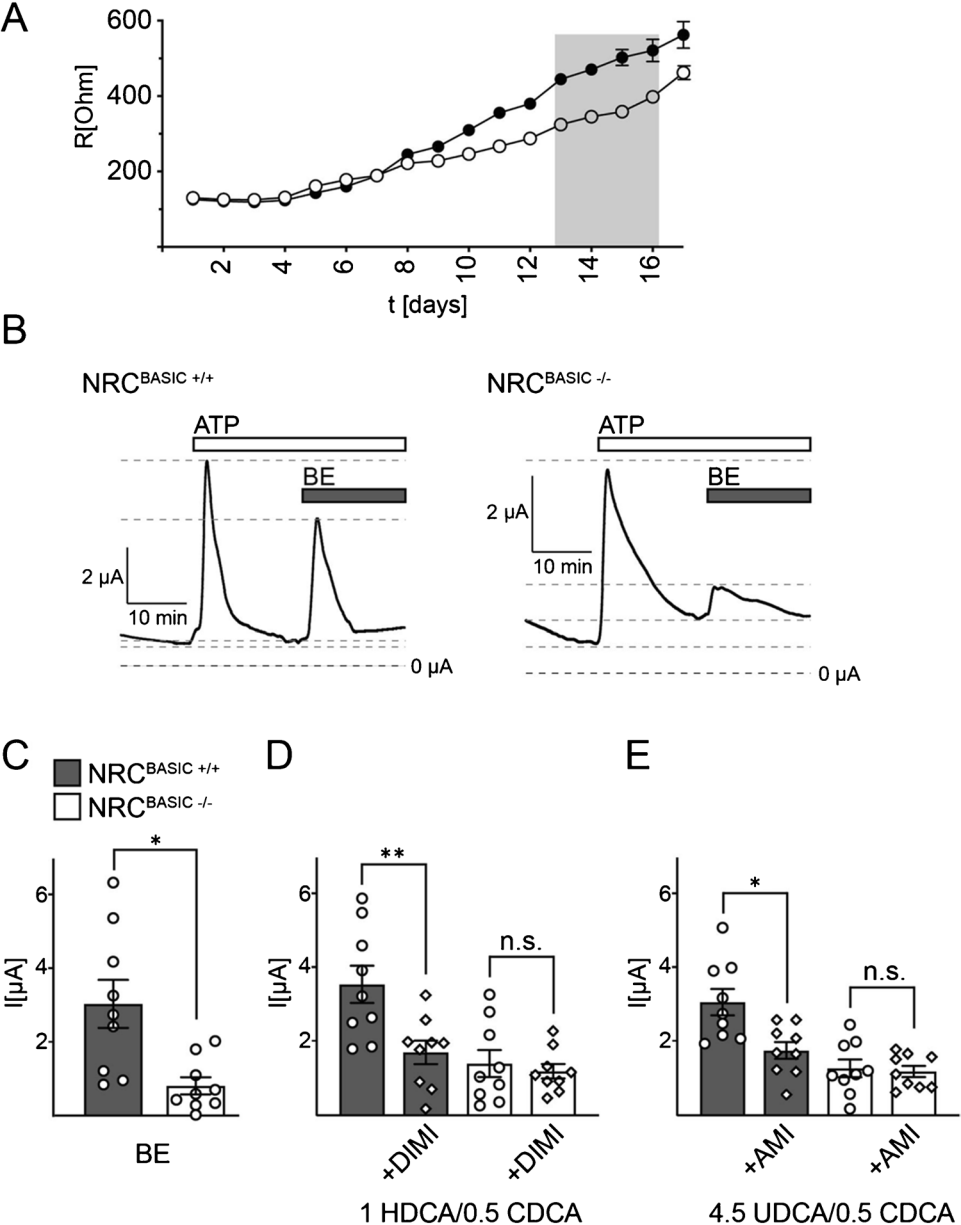
Bile acid-induced currents in normal rat cholangiocytes are mediated partly by BASIC. **A** Average development of the transepithelial electrical resistance (TEER) of NRC^BASIC+/+^ (closed circles, data from Fig. 1B) and NRC^BASIC−/−^ (open circles) epithelial layers over time. Error bars represent the SEM, *n* = 63. **B** Representative traces of transepithelial currents recorded from NRC^BASIC+/+^ (left panel) and NRC^BASIC−/−^ (right panel). ATP (100 µM) and BE (30 mg/ml) were applied to the apical lumen at the indicated times. **C** Quantitative comparison of the peak amplitudes of the transepithelial current increases induced by apical application of BE to NRC^BASIC+/+^ and NRC^BASIC−/−^. **D** Quantitative comparison of the peak amplitudes of the transepithelial current increases induced by apical application of 1 mM HDCA and 0.5 mM CDCA to NRC^BASIC+/+^ and NRC^BASIC−/−^ in the absence or presence of 10 µM diminazene (+ DIMI). **E** As in **D** but with 1 mM UDCA and 0.5 mM CDCA and in the absence or presence of 10 µM amiloride (+ AMI). Error bars = SEM, *n* = 9, * indicates a significant difference to control at a level of *p* < 0.05 or ** *p* < 0.01

The NRC^BASIC−/−^ cells showed a normal cholangiocyte phenotype and a similar growth rate as NRC^BASIC+/+^ cells. Of note, the development of the transepithelial electrical resistance of NRC^BASIC−/−^ cells was only similar to NRC WT cells during the first 8 days but subsequently did not increase to the same extent (Fig. 4A). At days 13–16, the TEER of NRC^BASIC−/−^ cells was approximately 125 Ω/cm^2^ lower compared with NRC^BASIC+/+^ cells. This result indicates a putative role for BASIC under basal secretory conditions of cholangiocytes.

Next, we compared the effect of bile extract (30 mg/ml) between NRC^BASIC−/−^ cells and NRC^BASIC+/+^ cells. While the response to ATP was similar in both cell lines, the response to BE was drastically decreased in NRC^BASIC−/−^ cells (NRC^BASIC+/+^ 3 ± 0.7 µA, NRC^BASIC−/−^ 0.8 ± 0.4 µA) (Fig. 4B/C). This is in line with our finding that diminazene inhibited BE-dependent currents and shows that a large portion of the bile acid-induced response in cholangiocytes is indeed mediated by BASIC. The response to co-application of 1 mM HDCA and 0.5 mM CDCA was similarly reduced in NRC^BASIC−/−^ cells (Fig. 4D/E). Next, we tested the inhibitory effect of diminazene and amiloride, another specific Deg/ENaC inhibitor, on bile acid-induced currents in NRC^BASIC−/−^ and NRC^BASIC+/+^ cells. While in NRC^BASIC+/+^ cells the presence of diminazene and amiloride decreased the bile acid-dependent current drastically in NRC^BASIC−/−^ cells, the bile acid-dependent current was not decreased by either diminazene or amiloride.

Taken together, these data from our pharmacological and BASIC knockout experiments show that in cholangiocytes, bile acids induce a current which is partly mediated by BASIC.

## Discussion

In recent years, some progress has been made in understanding how BASIC can be activated and what the underlying mechanism is. Moreover, pharmacological tools have been identified and the sites of expression were described in detail [37, 5, 38, 39, 30]. However, the physiological function of the channel remains puzzling. Bile acids have been shown to be natural activators of BASIC [38, 39] but whether this mode of activation is of any physiological relevance also remains an open question. Cholangiocytes are probably the cell type that is in contact with the highest possible concentrations of bile acids in the body. Here we show that in a cholangiocyte cell line, BASIC is partly responsible for bile acid-induced transepithelial currents, suggesting that it is involved in epithelial transport processes in bile ducts.

We have made two approaches to study the role of BASIC in cholangiocytes: We used pharmacological tools, namely bile acids as activators and diminazene and amiloride as inhibitors, and a CRISPR-cas9(D10A)-based knockout strategy to show that BASIC contributes to transepithelial currents elicited by bile acids. Application of millimolar concentrations of various bile acids induced transepithelial current, which is in line with previous findings [38], and diminazene and amiloride decreased these currents to approximately 50%. In heterologous expression systems, the onset of the BASIC response to bile acids is faster than in cholangiocytes, which may be explained by either a slower application of bile acids or additional cellular components that affect BASIC currents in these cells. Furthermore, the bile acid response in cholangiocytes is only transient, which is also in contrast to BASIC currents in heterologous expression systems [38]. It will be interesting to investigate these apparent differences in detail. Ablation of BASIC in cholangiocytes reduced the bile acid-induced currents to the same extent. These findings indicate that approximately 50% of this current is indeed conducted by BASIC. Furthermore, our results demonstrate that the bile acid-induced current is mainly carried by Na^+^, which is in line with a cation-selective channel like BASIC. The remaining 50% of the bile acid-mediated current is BASIC-independent and thus carried by other transport mechanisms. We speculate that for example the electrogenic absorption of glucose via the Na^+^-dependent glucose transporter SGLT1 [19] or the activation of Na^+^-conducting P2X receptors [9] may contribute to this bile acid-mediated current.

The main task of cholangiocytes is to guarantee a continuous flow of bile with all its compounds towards the gallbladder and the duodenum. This is achieved by secretion of HCO_3_^−^, which is followed by an osmotically driven efflux of water [32]. How does Na^+^ absorption fit into this picture? Interestingly, it was shown that ENaC is also expressed in mouse biliary epithelial cells. Here it absorbs Na^+^ upon increased mechanical stress induced by high bile flow rates, which leads to water absorption thereby decreasing bile flow again [23]. In a scenario where high bile flow with high concentrations of bile acids occurs, BASIC, as a sensor for high concentrations of bile acids, may aid ENaC in responding to a high bile flow and reduce flow by Na^+^ absorption and subsequent water reabsorption.

**Fig. 5:**
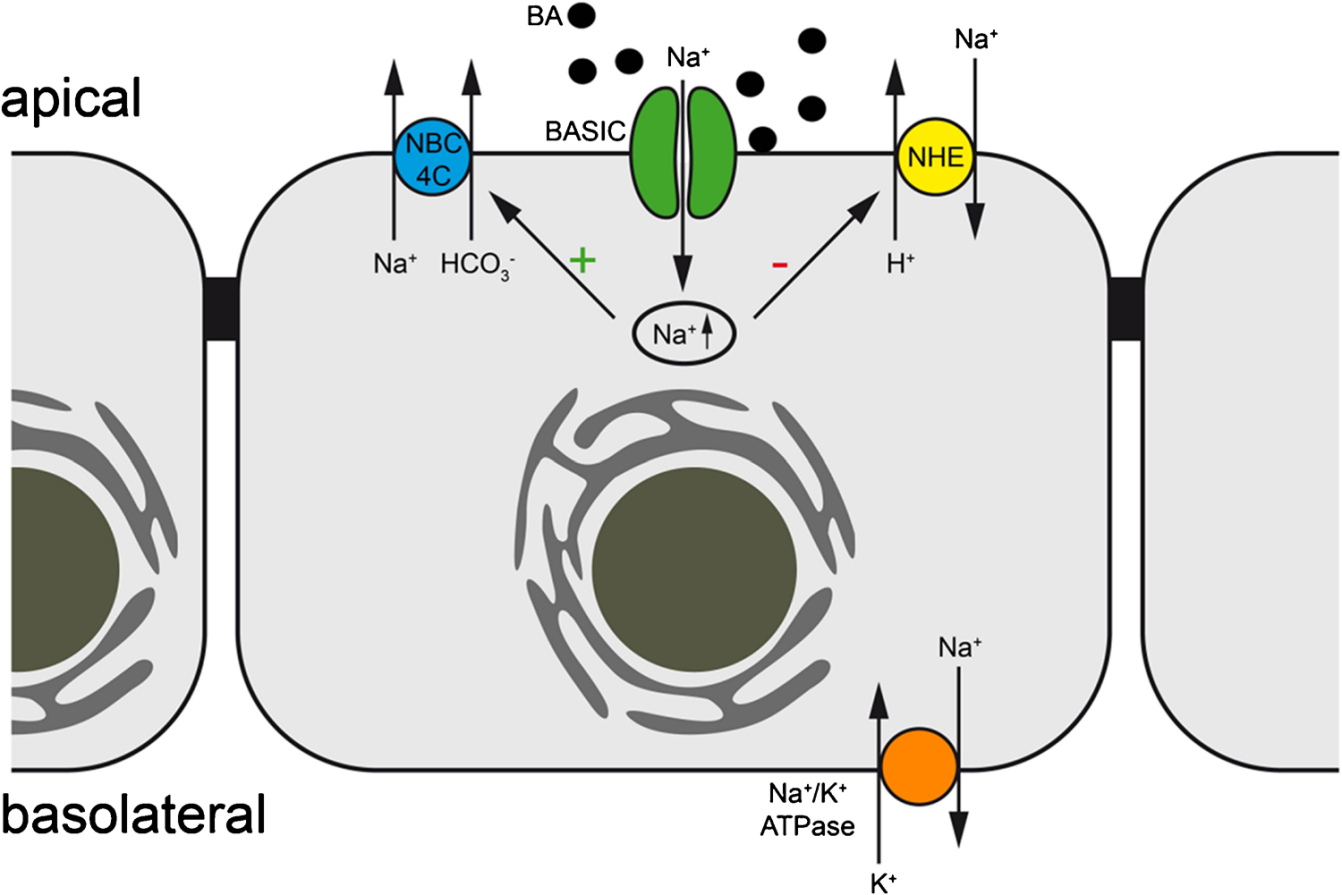
Possible role of BASIC in cholangiocyte ion transport. Bile acids (BA) activate BASIC. This leads to an increase of the intracellular Na^+^ concentration and in turn results in an increase in HCO_3_^−^ secretion via NBC4c and a decrease in H^+^ secretion via NHE

What other implications could a bile acid-dependent absorption of Na^+^ in cholangiocytes have? HCO_3_^−^ secretion is one of the most important tasks of cholangiocytes und guarantees an adequate bile flow [2, 13, 32]; furthermore, the secreted HCO_3_^−^ forms the so-called HCO_3_^−^ umbrella [14], which, together with the glycocalyx of cholangiocytes, is important for cholangiocyte protection as it keeps bile acids that are in close proximity to the cell membrane deprotonated and thus less toxic for the cells as polar bile acids cannot permeate through the cell membrane [14]. The anion exchanger 2 (AE2) is responsible for the vast majority of HCO_3_^−^ secretion in cholangiocytes and it uses the Cl^−^ gradient as a driving force [32]. In rat cholangiocytes, a subtype of Na^+^-HCO_3_^−^ cotransporter (NBC), NBC4c, was found at the apical membrane [1]. Expressed at the apical membrane, it could allow further HCO_3_^−^ secretion if enough intracellular Na^+^ would be present to drive Na^+^ and HCO_3_^−^ secretion. A BASIC-driven accumulation of Na^+^ at the intracellular side of the apical membrane could thus increase the secretion of HCO_3_^−^ (Fig. 5) and lead to a strengthening of the protective HCO_3_^−^ umbrella, which would be especially important if bile acid concentration in the bile ducts would increase.

Cholangiocytes also express different Na^+^-H^+^ exchanger (NHE) at their apical and basolateral membrane, which are crucial for maintaining the intracellular pH as they allow secretion of H^+^ in exchange with Na^+^ [31]. To guarantee the proper protective function of the HCO_3_^−^ umbrella, however, it is important to tightly control the apical H^+^ secretion via NHEs. A BASIC-mediated accumulation of Na^+^ in the apical compartment of the cell would decrease the driving force for H^+^ secretion by NHEs, when bile acid concentrations are high (Fig. 5), and would thus maintain the alkaline pH of the bile fluid and protect the epithelium from bile acids. This could represent another possible role for BASIC in cholangiocyte physiology.

Small amounts of bile acids can be reabsorbed by cholangiocytes from intrahepatic bile ducts and returned back to hepatocytes via the cholehepatic shunt pathway, which provides a recycling pathway for bile acids and may be involved in several processes where bile acids play a role e.g. gene expression, second messenger regulation, and modulation of ion transport processes [32, 42]. While unconjugated bile acids can passively diffuse through the apical membrane of cholangiocytes, conjugated bile acids are reabsorbed by the Na^+^-dependent bile acid transporter SLC10A2 [20]. We speculate that the bile acid-dependent increase in Na^+^ absorption mediated by BASIC may decrease the driving force for Na^+^ and consequently lower the amount of bile acids that can be reabsorbed via SLC10A2. Thus, BASIC might affect indirectly the cholehepatic shunt pathway.

Taken together, our data suggest that BASIC plays a regulatory role in fine-tuning ion transport by rat cholangiocytes.

## Data Availability

This manuscript has no associated data. All data are contained within the manuscript. Cell lines are available upon request.
